# Cadmium-Induced Hydrogen Accumulation Is Involved in Cadmium Tolerance in *Brassica campestris* by Reestablishment of Reduced Glutathione Homeostasis

**DOI:** 10.1371/journal.pone.0139956

**Published:** 2015-10-07

**Authors:** Qi Wu, Nana Su, Qin Chen, Wenbiao Shen, Zhenguo Shen, Yan Xia, Jin Cui

**Affiliations:** College of Life Sciences, Laboratory of Nanjing Agricultural University, Nanjing, Jiangsu Province, China; Zhejiang University, CHINA

## Abstract

Hydrogen gas (H_2_) was recently proposed as a therapeutic antioxidant and signaling molecule in clinical trials. However, the underlying physiological roles of H_2_ in plants remain unclear. In the present study, hydrogen-rich water (HRW) was used to characterize the physiological roles of H_2_ in enhancing the tolerance of *Brassica campestris* against cadmium (Cd). The results showed that both 50 μM CdCl_2_ and 50%-saturated HRW induced an increase of endogenous H_2_ in *Brassica campestris* seedlings, and HRW alleviated Cd toxicity related to growth inhibition and oxidative damage. Seedlings supplied with HRW exhibited increased root length and reduced lipid peroxidation, similar to plants receiving GSH post-treatment. Additionally, seedlings post-treated with HRW accumulated higher levels of reduced glutathione (GSH) and ascorbic acid (AsA) and showed increased GST and GPX activities in roots. Molecular evidence illustrated that the expression of genes such as *GS*, *GR1* and *GR2*, which were down-regulated following the addition of Cd, GSH or BSO, could be reversed to varying degrees by the addition of HRW. Based on these results, it could be proposed that H_2_ might be an important regulator for enhancing the tolerance of *Brassica campestris* seedlings against Cd, mainly by governing reduced glutathione homeostasis.

## Introduction

Cadmium (Cd) is a highly water-soluble toxic heavy metal that can be quickly taken up by plant roots in soil, where it can be consumed by animals, including humans, through the food chain [1, 2]. In plants, Cd accumulation has been suggested to directly and/or indirectly inhibit physiological processes, such as photosynthesis and respiration, alter ultrastructural characteristics and affect the nutritional status [3–5]. As the aggravation of industrial and agricultural pollution, Cd-induced reduction in the yield of crops and the downgrade of their quality is becoming more and more severe, which leads to a wide range of investigation in possible methods of alleviating Cd toxicity in plants, especially in plant growth regulators, such as 5-aminole vulinic acid, polyamines and malic acid [6, 7].

Recently, using exogenous hydrogen-rich water (HRW, a kind of hydrogen gas donor) in plants has been suggested an effective way of alleviation against Cd toxicity (Cui et al., Wu et al), and this effect is mainly due to the H_2_-induced increase of antioxidant ability in plants. In addition, H_2_ has been also found to be a beneficial gaseous molecule in alleviating other abiotic stresses, including high salinity [8], paraquat toxicity [9] and aluminum stress [10, 11]. Moreover, H_2_ has been shown to delay postharvest ripening and senescence of kiwifruit [12], regulate cucumber adventitious root development [13] and promote anthocyanin synthesis in radish sprouts [14]. H_2_ metabolism in bacteria and algae has long been studied, and the hydrogenase system has been elucidated [15–17]. However, despite a demonstrated release of H_2_ [18, 19], it remains unclear whether the hydrogenase system is present in higher plants. Furthermore, few studies have addressed the biological effects of H_2_ or provided experimental evidence for H_2_ as a bio-effecter or signaling molecule in plants.

It has been confirmed that Cd-induced excess reactive oxygen species (ROS) is one of the biggest causes of plant damnification, which causes oxidative damage to lipids, proteins and nucleic acids, and leads to detrimental effects on normal metabolism [20, 21]. To reduce the oxidative damage and acclimate the stressful environment, plants produce a variety of enzymatic and small molecules to tightly regulate the ROS homeostasis.

Glutathione (GSH) is one kind of plant metabolites that function as antioxidants in plants and in animals that consume them. The synthesis of GSH involves the enzymes γ-glutamylcysteine synthase (γ-GCS) and glutathione synthase (GS) [22]. GSH synthesis is inhibited by buthionine sulfoximine (BSO), an inhibitor of γ-GCS. In plants, GSH plays a central role in the cellular defense against environmental toxins. First, as an antioxidant, GSH can act directly to reduce most ROS generated during stress, resulting in the oxidation of GSH to glutathione disulfide (GSSG) [23, 24]. Changes in the ratio of the reduced (GSH) to oxidized (GSSG) form during the degradation of H_2_O_2_ are important in certain redox signaling pathways [25]. Second, GSH contributes to the biosynthesis of ascorbate (AsA) through the ascorbate-glutathione cycle (AsA-GSH cycle) in which dehydroascorbate (oxidized ascorbate, DHA) is reduced to AsA using electrons from GSH [26]. Third, GSH has been shown to be a precursor of phytochelatins (PCs), which are a set of novel heavy metal-binding peptides found in plants [27]. In addition, GSH is a substrate of glutathione peroxidase (GPX) and glutathione-S-transferase (GST), both of which are involved in the removal of ROS and peroxides [28].

Our recent study showed that pretreatment with HRW could alleviate Cd toxicity and influence GSH synthesis in *Medicago sativa* [10], which promoted us to propose whether there is a link between H_2_ and GSH in alleviating Cd stress. However, additional details regarding the role of GSH in HRW-induced alleviation of Cd stress have not been reported. In this work, *Brassica campestris* was selected to test the biological effects of H_2_. *Brassica campestris* is one of the most widely grown vegetables in the world and Brassica vegetables are highly regarded for their nutritional value including high amounts of vitamin C, soluble fiber and multiple nutrients with potent anticancer properties, while its productivity and quality are considerably decreased due to abiotic stresses, including Cd exposure. In addition, we studied the effect of HRW on the alleviation of plant growth inhibition caused by CdCl_2_ and examined the links between endogenous GSH metabolism and the HRW-triggered improvement of inhibition in seedlings challenged with Cd. Furthermore, we investigated the influence of CdCl_2_ on H_2_ production in *Brassica campestris* seedlings. This information is beneficial to our better understanding of the mechanisms involved in the defense of plant cells against Cd stress mediated by H_2_.

## Materials and Methods

### Plant materials, growth conditions, and treatments

*Brassica campestris* seeds (*Brassica campestris* spp. *chinensis* L., Dongfang 2), kindly supplied by the Jiangsu Academy of Agricultural Sciences (Jiangsu Province, China), were surface-sterilized with 5% NaClO for 5 min and then rinsed extensively in distilled water before germination on moist gauze for 1 d at 23^◦^C in the darkness. Uniform-sized seeds were selected and transferred to plastic chambers containing quarter-strength Hoagland’s solution. After growth in nutrient solution for one day, seedlings were transferred into quarter-strength Hoagland’s solutions with 0 or 50 μM CdCl_2_ and incubated for another 12 h. Our preliminary Cd concentration screening experiments showed that fresh weight and root length of cabbage seedlings grown at 50 μM Cd were reduced by 50% compared with the control (no Cd added)[29]. Therefore, we applied this Cd concentration in this study. Moreover, in order to explore the effects of HRW on alleviating Cd toxicity, we treated the seedlings with different HRW concentrations, and all the related indexes demonstrated that the treatment with 50% saturated HRW produced the maximal alleviating effects on Cd stress [29]. Therefore, after the removal of cadmium stress, the seedlings were incubated in quarter-strength Hoagland’s solutions with or without 50% saturated HRW. In addition, 10 μM GSH and 100 μM BSO were also set as treatments to investigate the link between the HRW and GSH. The concentrations of GSH and BSO were initially determined following the methods described by Chen et al [30] with some modifications.

We described these different treatments as (I) H_2_O→H_2_O, (II) H_2_O→HRW, (III) Cd→H_2_O, (IV) Cd→HRW, (V) Cd→GSH + H_2_O, (VI) Cd→GSH + HRW, (VII) Cd→BSO + H_2_O, and (VIII) Cd→BSO + HRW. All treatment solutions were renewed every 12 h to maintain constant concentrations. The pH of solution was maintained at 6.0, and the relative humidity was approximately 65–75%. Seedlings were grown in an illuminated incubator (12 h light with a light intensity of 200 ± 5 μmol·m^-2^·s^-1^, 25 ± 1^◦^C, and 12 h dark, 23 ± 1^°^C). After various treatments, the seedlings were sampled and growth parameters were determined. Root tissues were used immediately or frozen in liquid nitrogen for further analysis.

### Preparation of hydrogen-rich water (HRW)

Purified H_2_ gas (99.99%, *v/v*) was generated using an H_2_-producing apparatus (SCH-500, Saikesaisi Hydrogen Energy Co. Ltd., Shandong, China). Purified H_2_ gas was bubbled into 4,000 mL of distilled water (pH 6.0, 25^°^C) at a rate of 160 mL·min^-1^ for 2 h, which was a sufficient duration to saturate the solution with H_2_ [11]. The corresponding HRW was then immediately diluted to a 50% concentration (*v/v*).

### Determination of endogenous H_2_ concentrations

Headspace samples of gas were collected to analyze endogenous H_2_ concentrations using gas chromatography (GC). Approximately 1 g of *Brassica campestris* seedlings was placed in a vial, and pure nitrogen gas was then bubbled into the vial to displace the air. Subsequently, the vial was immediately capped and incubated at room temperature for 12 h before the headspace was analyzed by GC. The chromatographic system (GC-2014; Shimadzu, Ltd., Kyoto, Japan) consisted of a gas chromatograph equipped with a thermal conductivity detector (TCD) and a column containing Molecular Sieve 5 Å stationary phase (Shimadzu, Ltd, Kyoto, Japan). The bridge current was 80 mA, and the column temperature was held constant at 50°C. The injection and detector temperatures were adjusted to 120 and 80°C, respectively. Nitrogen gas was used as the carrier gas, and the air pressure was 0.5 MPa.

### Determination of Cd concentrations in plant tissues

At harvest, one hundred plant samples from each treatment were collected and separated into root, stem and leaf fractions. The samples were then carefully washed with deionized water after rinsing with 20 mM EDTA-Na solution for approximately 30 min, dried at 105°C for 20 min, and then held at 70°C in an oven until completely dried [31]. The dried plant samples were ground to a powder and digested in a solution containing an 87:13 HNO_3_:HClO_4_ solution. The concentrations of cadmium were determined using an atomic absorption spectrophotometer (180–80 Hitachi, Tokyo, Japan) as described by Liu et al [32].

### Histochemical analysis

Stress-induced generation of O_2_^-^ *in situ* was detected by nitroblue tetrazolium (NBT) staining [33]. The roots which were not cut from the seedlings were immersed in a 0.1% solution of NBT in 10 mM potassium phosphate buffer (pH 7.8) containing 10 mM sodium azide (NaN_3_) and then incubated in the dark at 22°C for 10 min until a purple-blue color became visible. Hydrogen peroxide (H_2_O_2_) production was detected by staining with a freshly prepared 3, 3′-diaminobenzidine (DAB) solution (0.1% *w/v*, pH 3.8) for 1 h [34]. After extensive washing, all of the decolorized roots were observed under a light microscope (model Stemi 2000-C; Carl Zeiss, Jena, Germany) and photographed using color film (Powershot A620; Canon Photo Film, Tokyo, Japan). About 20 seedlings with roots were collected for each experiment and three independent repetitions were performed.

### Determination of TBARS concentrations

Lipid peroxidation based on thiobarbituric acid reactive substances (TBARS) reported in malondialdehyde (MDA) equivalents was used as an indicator of oxidant stress in Cd-exposed roots. Lipid peroxidation was estimated by measuring the concentration of TBARS as described by Jin et al [9]. Briefly, fresh root tissues (0.5 g) were homogenized in a mortar with 5 mL of a solution containing 0.25% 2-thiobarbituric acid (TBA) and 10% trichloroacetic acid (TCA). The mixture was heated at 95°C for 30 min followed by rapid cooling in an ice bath and then centrifuged at 10,000×g for 10 min. The absorbance of the supernatant was read at 532 nm and corrected for nonspecific turbidity by subtracting the absorbance at 600 nm. The blank was 0.25% TBA in 10% TCA.

#### Determination of glutathione by fluorescence microscopy

The roots which were not cut from the seedling were exposed to 50 μM monochlorobimane (MCB) for 15 min to detect the level of endogenous glutathione [35, 36]. After removal of the staining solution, the preparation was thoroughly washed with phosphate buffer (pH 7.2) for 30 s to remove excess staining solution and then analyzed microscopically. Images of the fluorescent signal were captured using a digital camera mounted on a fluorescence microscope (Axio Imager A1, Carl Zeiss, Germany; excitation 365 nm), and the fluorescence intensity was calculated using Image-Pro Plus software (Media Cybenetics, USA). Results from four representative experiments are presented. About 15 seedlings with roots were collected for each experiment and two independent repetitions were performed for the determination of GSH.

### Enzyme activity assays

Approximately 0.5 g of frozen root tissue was extracted with 5 mL of extraction buffer containing 50 mM potassium phosphate (pH 7.5), 10 mM KCl, 1 mM EDTA, 5 mM dithiothreitol (DTT), 0.5 mM AEBSF (a protease inhibitor) and 1:4 (w/w) polyvinyl-poly-pyrrolidone (insoluble PVPP). The homogenates were centrifuged for 20 min at 12, 000 rpm, and the supernatants were used for enzyme assays.

The activity of GST was measured as described by Xia et al [37], with a modification in recording the absorbance at 412 nm. The activity of GR was assayed based the rate at which the absorbance at 340 nm decreased, according to the method of Cakmak and Marschner [38]. Glutathione peroxidase (GPX) activity was determined according to the modified method of Pailan et al [39]. GS activity was measured spectrophotometrically at 500 nm by the transferase assay described by Ortega et al [40]. GCS activity was measured as the rate of γ-EC formation at 30°C, as previously described [41].

### Measurement of glutathione and ascorbate levels

The levels of GSH and GSSG were determined using the enzyme-recycling method of Rahman et al [42] with certain modifications. To determine GSH and GSSG levels in roots, 0.3 g of frozen root tissue was homogenized and extracted in 2 mL of 0.1 M sodium phosphate buffer containing 5 mM EDTA (pH 7.5). After micro-centrifugation (20 min, 12000×g), total glutathione was measured in the supernatant. The assay was based on the sequential oxidation of GSH by 5,5’-dithiobis-2-nitrobenzoic acid (DTNB) and reduction of GSSG in the presence of NADPH and glutathione reductase.

Reduced ascorbate (AsA) and dehydroascorbate (DHA) were determined by the method of Srivastava et al [43]. This method is based on the reduction of Fe^3+^ to Fe^2+^ with ascorbic acid in an acid solution followed by the formation of a red chelate between Fe^2+^ and 2,20-bipyridyl. Treated and untreated root tissues were homogenized in 5% (w/v) m-phosphoric acid using a mortar and pestle under cool conditions. The homogenate was centrifuged at 12,000×g for 15 min. Total ascorbate was determined in a reaction mixture consisting of 0.2 mL of supernatant, 0.5 mL of 150 mM potassium phosphate buffer (pH 7.4) containing 5 mM EDTA, and 0.1 mL of 10 mM dithiothreitol (DTT) to reduce DHA into AsA. Reduced ascorbate was assayed in a similar manner except that DTT was substituted by 0.2 mL deionized H_2_O. Dehydroascorbate was determined by subtracting AsA from AsA + DHA. Ascorbate concentrations were calculated according to the standard curve prepared using L-ascorbic acid.

### Real-time quantitative RT-PCR analysis

Total RNA was isolated from root tissues using TRIZOL extraction reagent (Invitrogen, Gaithersburg, MD, USA). RNA purity was verified based on the ratio (>1.9) of 260/280 nm absorbance. DNA-free total RNA (5 μg) from different treatments was used for first-strand cDNA synthesis in a 20 μL reaction volume (Thermo Scientific, MD, Lithuania) according to the manufacturer’s instructions. Real-time quantitative PCR reactions were performed using a Mastercycler® ep realplex real-time PCR system (ABI7500, MD, USA) with Bestar^®^ SybrGreen qPCR mastermix (DBI, Bioscience Inc., Germany) in a 20 μL reaction volume according to the user manual.

PCR primers targeting *GCS*, *GS*, *GR1*, *GR2*, *GPX* and *GST* were designed using Primer Express®version3.0 (Applied Biosystems), and *actin* was designed following Xiao et al [44]. All primers (S1 Table) were synthesized by Genewiz Bio-engineering Ltd. Company (Suzhou, China). The expression levels are presented relative to those of corresponding control samples at the indicated time after normalization to *actin* transcript levels.

### Data presentation and statistical analysis

Values are shown as the mean ± SD of three independent experiments with three replicates each. Differences among treatments were analyzed by one-way analysis of variance (ANOVA) combined with Duncan’s multiple range test at a probability of *P* < 0.05.

## Results

### Cadmium stimulated H_2_ production

**Fig 1:**
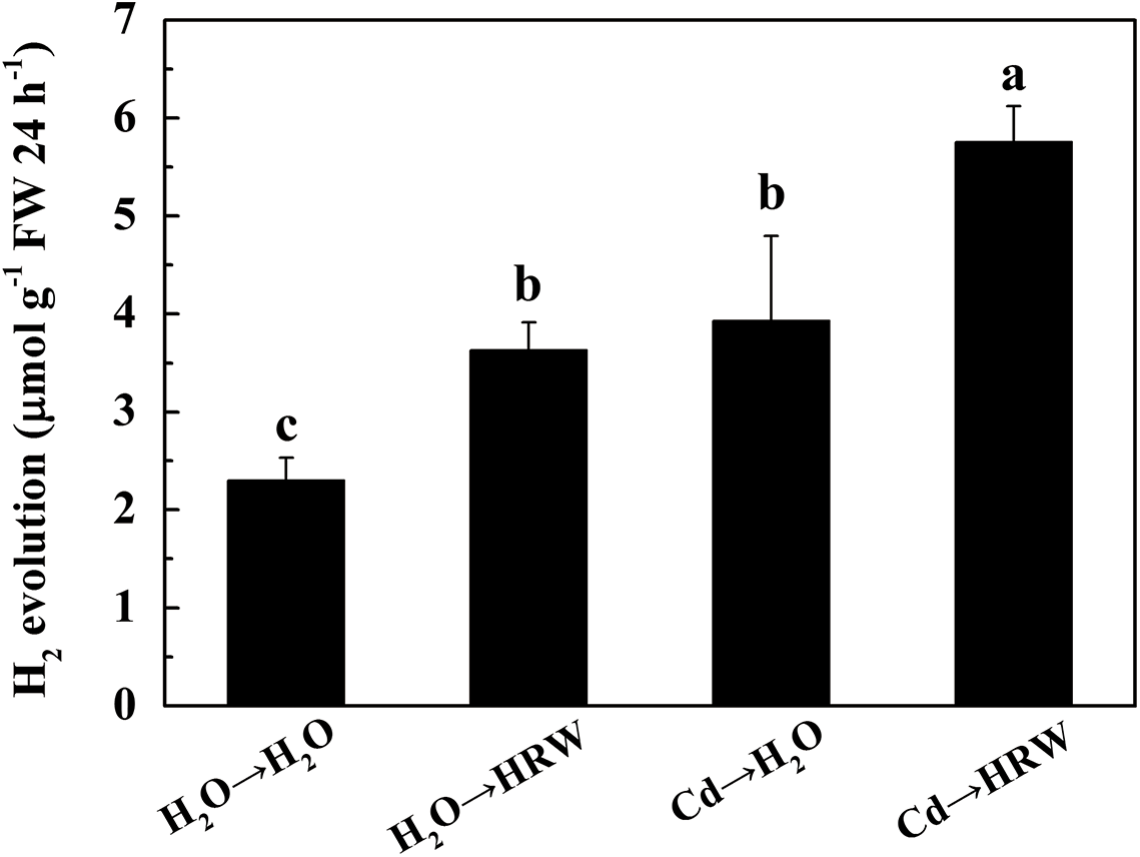
An increase in hydrogen (H_2_) evolution in *Brassica campestris* seedlings upon CdCl_2_ and HRW treatment. The germinated seeds were grown in nutrient solution for one day and then shifted to solutions with 0 or 50 μM CdCl_2_ for another 12 h. After the removal of Cd stresses, the seedlings were then incubated in solution with H_2_O or 50% saturation of HRW for 48 h. Data are means ± SE from three independent experiments. Bars with different letters are significantly different at *P*<0.05 according to Duncan’s multiple range test.

To investigate whether Cd affects endogenous H_2_ production, gas chromatography was applied to monitor the release of H_2_. As shown in Fig 1, H_2_ production was significantly increased in seedlings upon treatment with 50 μM CdCl_2_ compared to the corresponding control (H_2_O→H_2_O). Under non-stress conditions, a significant increase in H_2_ production by post-treatment of HRW was observed. Moreover, in comparison with the plants challenged with Cd alone, HRW treatment of seedlings after Cd stress increased H_2_ production by approximately 46%.

### HRW alleviated the inhibitory effects of Cd on seedling growth

**Fig 2:**
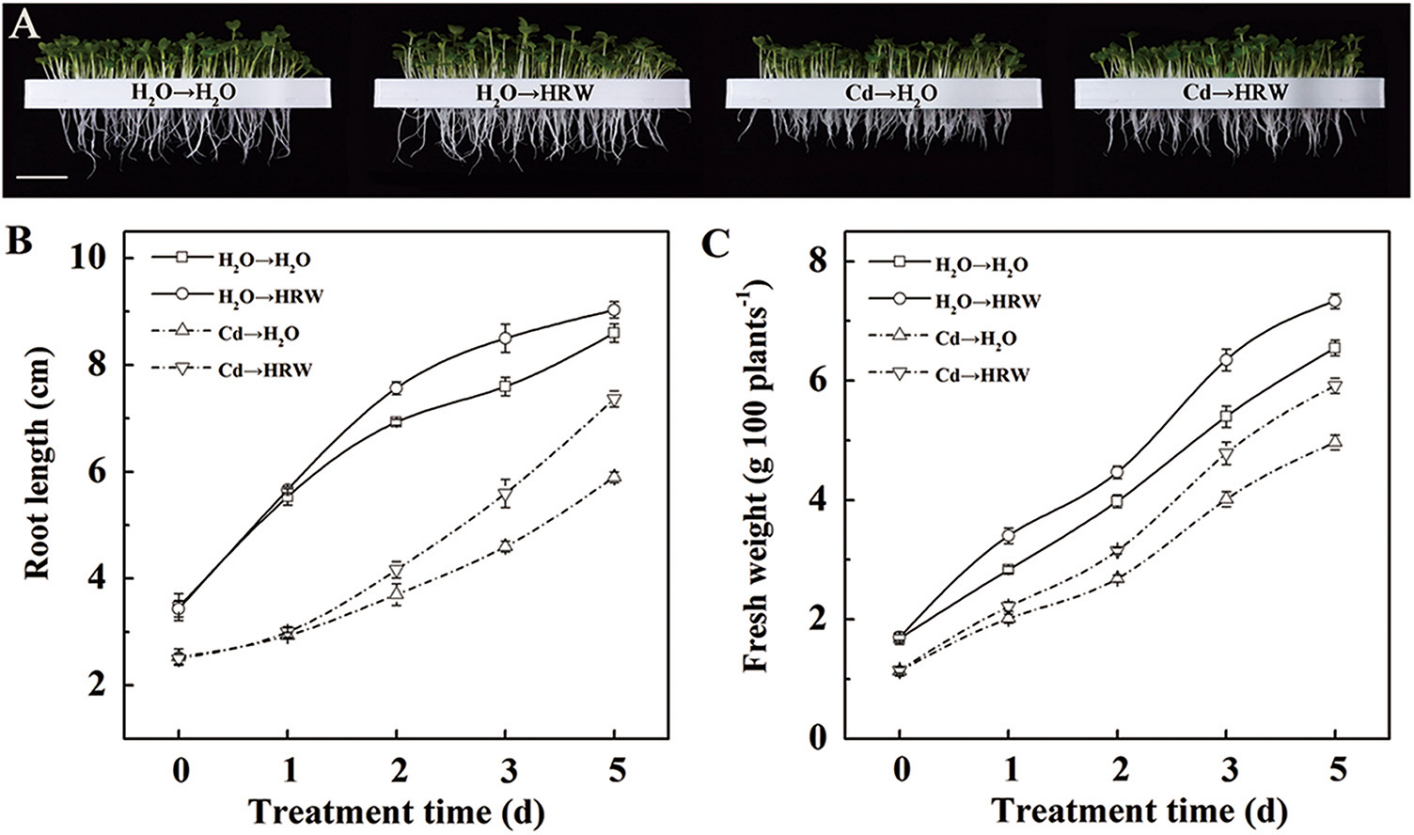
HRW post-treatment alleviated Cd stress-induced *Brassica campestris* seedlings growth inhibition. The germinated seeds were grown in nutrient solution for one day and then shifted to solutions with 0 or 50 μM CdCl_2_ for another 12 h. After the removal of Cd stresses, the seedlings were then incubated in solution with H_2_O or 50% saturation of HRW for 5 days. Bar = 3 cm (A). Changes in root length (B) and fresh weight (C) during the 5 days post-treated with H_2_O or 50% saturation of HRW. Data are means ± SE from three independent experiments. Bars with different letters are significantly different at *P*<0.05 according to Duncan’s multiple range test.

Changes in root length and fresh weight during 5 days of treatment are shown in Fig 2. Seedlings showed a rapid increase in root length and fresh weight during the 5 days of cultivation under normal conditions, and the root length and fresh weight after 2 days of incubation in HRW were higher than those in H_2_O. Treatment with HRW considerably increased the root length and fresh weight regardless of Cd stress. This difference became more distinct with prolonged incubation time.

Cd levels in different parts of the seedlings were determined to confirm whether HRW affects Cd translocation from root to shoot. As expected, the Cd concentration decreased in roots and increased in shoots over time. However, no variation in the Cd concentrations of roots or shoots was observed between the HRW and H_2_O post-treatments (S1 Fig).

### HRW enhanced the synthesis of GSH in roots

**Fig 3:**
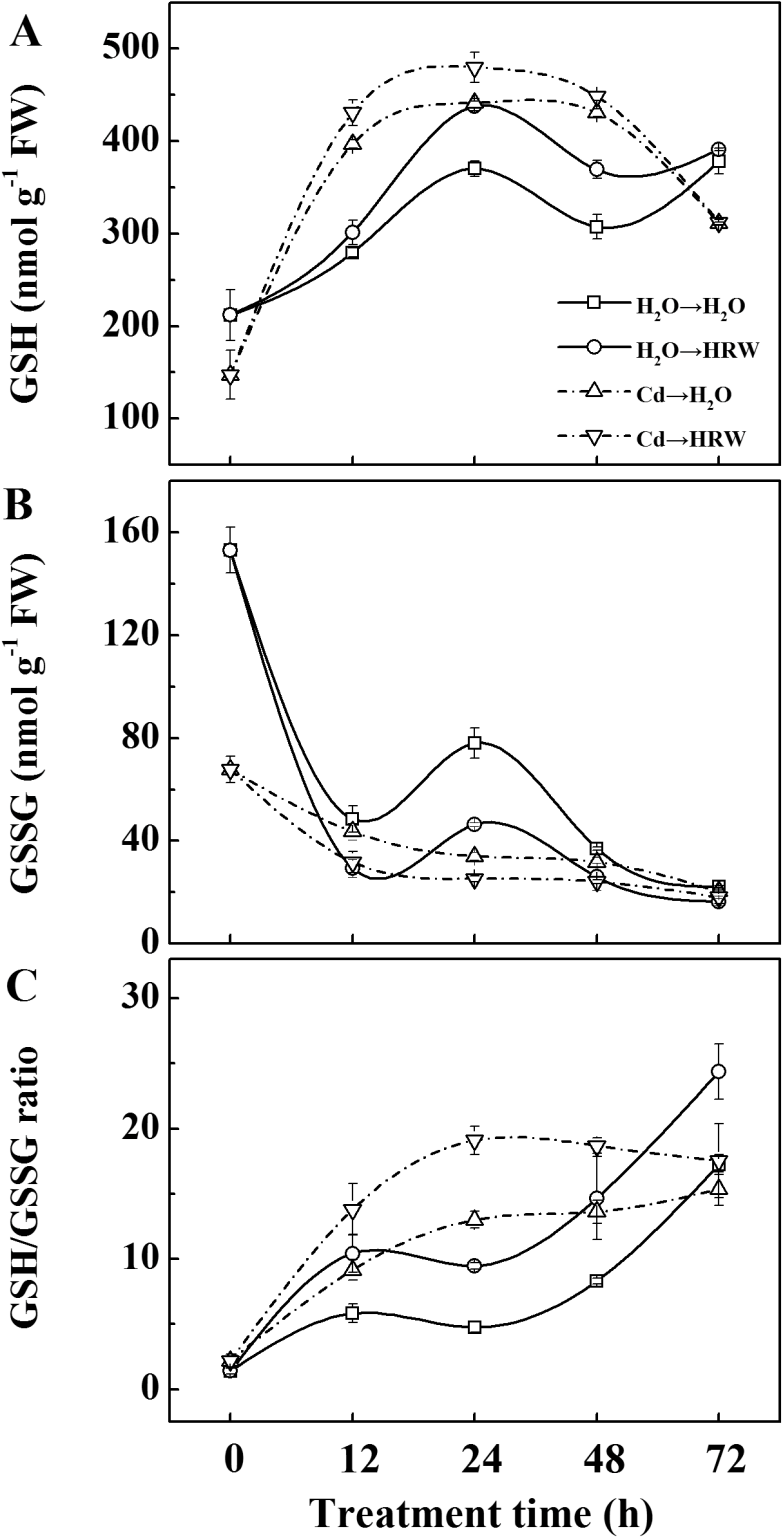
Time course changes of GSH pool in roots upon Cd stress and HRW post-treatment. The germinated seeds were grown in nutrient solution for one day and then shifted to solutions with 0 or 50 μM CdCl_2_ for another 12 h. After the removal of Cd stresses, the seedlings were then incubated in solution with H_2_O or 50% saturation of HRW for 72 h. Concentration of GSH (A), GSSG (B) and the ratio of GSH/GSSG (C) in root tissues were detected at the indicated time points of treatments. Data are means ± SE from three independent experiments. Bars with different letters are significantly different at *P*<0.05 according to Duncan’s multiple range test.

To determine whether HRW promotes GSH synthesis in roots, the GSH concentration was examined during a 72-h treatment (Fig 3). Initially, lower GSH and higher GSSG concentrations were observed in Cd treatments compared with non-Cd treatments. However, there was a very rapid induction of GSH synthesis in samples treated with Cd, especially during the first 12 h. This rapid GSH synthesis led to a much higher GSH value within the next 24 h. The GSH value reached a maximum level at 24 h for all treatments and then decreased until 48 h. More importantly, at 12, 24 and 48 h, a higher GSH concentration and a higher GSH/GSSG ratio were recorded in samples post-treated with HRW compared with corresponding treatments, regardless of Cd exposure. The GSSG concentration showed a slight decrease under Cd stress over time, whereas there was a sinusoidal trend in the non-Cd treatments, with higher values at 0 and 24 h.

### Effects of GSH, BSO and HRW on Cd-induced inhibition of root elongation and oxidative damage

**Fig 4:**
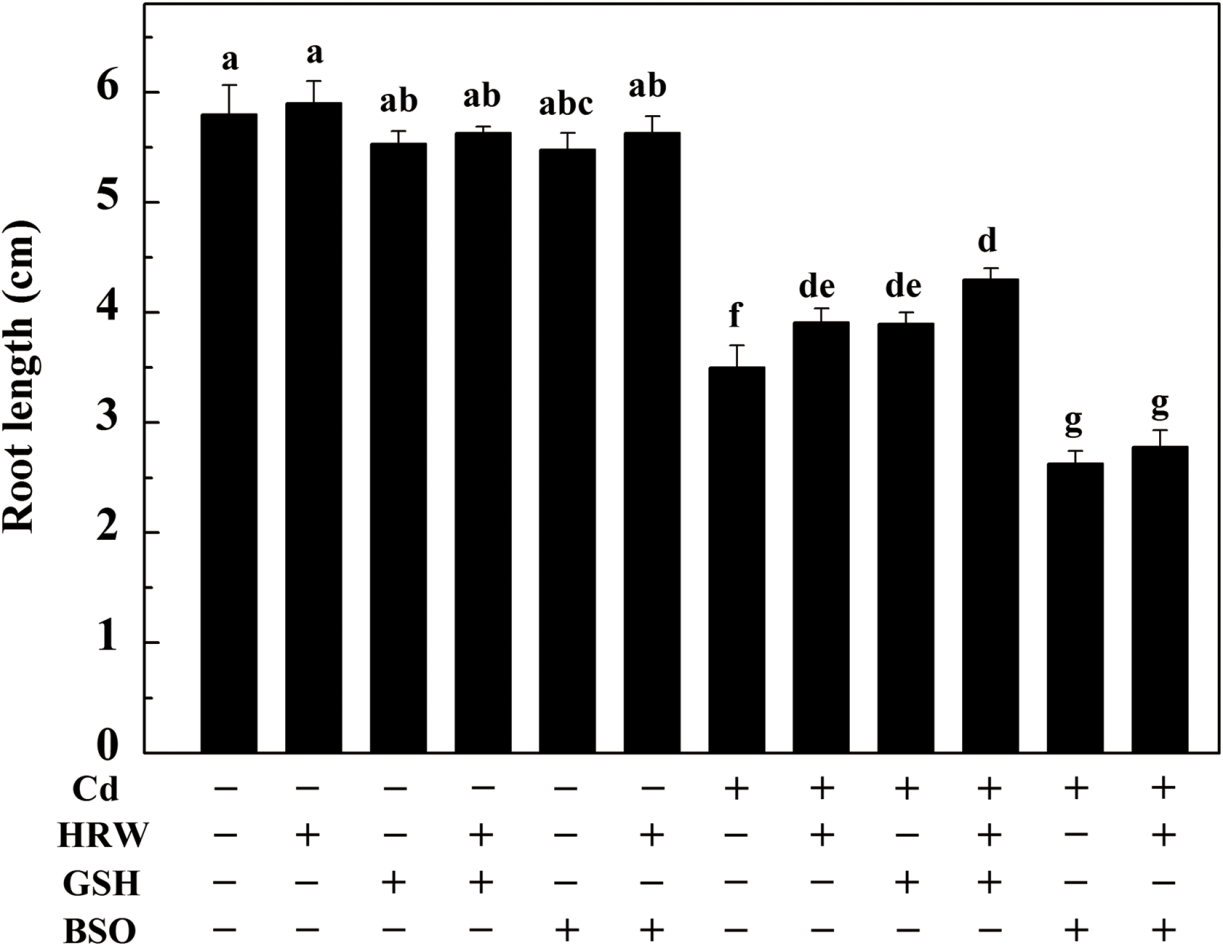
Effects of GSH, BSO and HRW alone or their combinations on Cd-induced inhibition of root elongation in *Brassica campestris* seedlings. The germinated seeds were grown in nutrient solution for one day and then shifted to solutions with 0 or 50 μM CdCl_2_ for another 12 h. After the removal of Cd stresses, the seedlings were then incubated in solution with 25 μM GSH, 10 μM BSO or 50% saturation of HRW alone or their combinations for 48 h. Data are means ± SE from three independent experiments. Bars with different letters are significantly different at *P*<0.05 according to Duncan’s multiple range test.

The root length and substances related to oxidative stress were measured to investigate the link between GSH biosynthesis and HRW-induced alleviation of root growth and oxidative stress. As shown in Fig 4, treatment with Cd for 12 h markedly inhibited root elongation. However, no significant difference in root length was observed in the non-Cd treatment, although the values were reduced due to GSH and BSO addition. In comparison with samples subjected to Cd treatment alone, HRW or GSH post-treatment for 48 h significantly increased the root length. This increase in root length was enhanced when HRW and GSH were added together. We also noted that the addition of a GSH synthesis inhibitor, BSO, after Cd treatment with Cd could further inhibit root elongation. Interestingly, this BSO response could not be obviously reversed by HRW addition.

**Fig 5:**
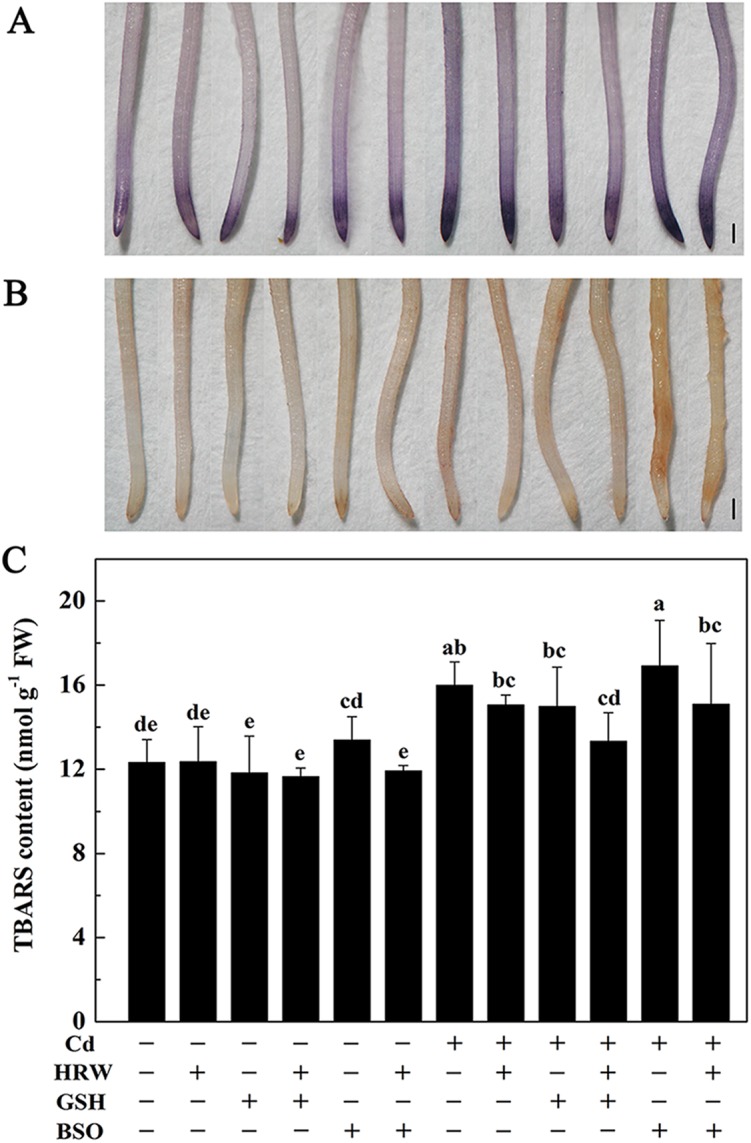
Effects of GSH, BSO and HRW alone or their combinations on loss of plasma membrane integrity (A), reactive oxygen species (ROS) localization (B) and TBARS (C) in the roots of *Brassica campestris* under Cd or none stress. The germinated seeds were grown in nutrient solution for one day and then shifted to solutions with 0 or 50 μM CdCl_2_ for another 12 h. After the removal of Cd stresses, the seedlings were then incubated in solution with 25 μM GSH, 10 μM BSO or 50% saturation of HRW alone or their combinations for 48 h. Data are means ± SE from three independent experiments. Bars with different letters are significantly different at *P*<0.05 according to Duncan’s multiple range test.

Subsequently, we performed histochemical analysis of O_2_^-^ and H_2_O_2_ and determined the TBARS concentration. As expected, low production of O_2_^-^ (NBT staining) and H_2_O_2_ (DAB staining) were detected in Cd-free plants, whereas those treated with Cd exhibited heavier staining results (Fig 5A and 5B). When HRW or GSH was applied, the heavy staining in the Cd-stressed roots was reduced. Additionally, the addition of BSO after Cd resulted in maximal staining, whereas reduced staining was observed with BSO treatment with HRW. Changes in TBARS concentration exhibited a similar trend (Fig 5C).

### Effects of GSH, BSO and HRW on the concentration of GSH in *Brassica campestris* seedling roots

**Fig 6:**
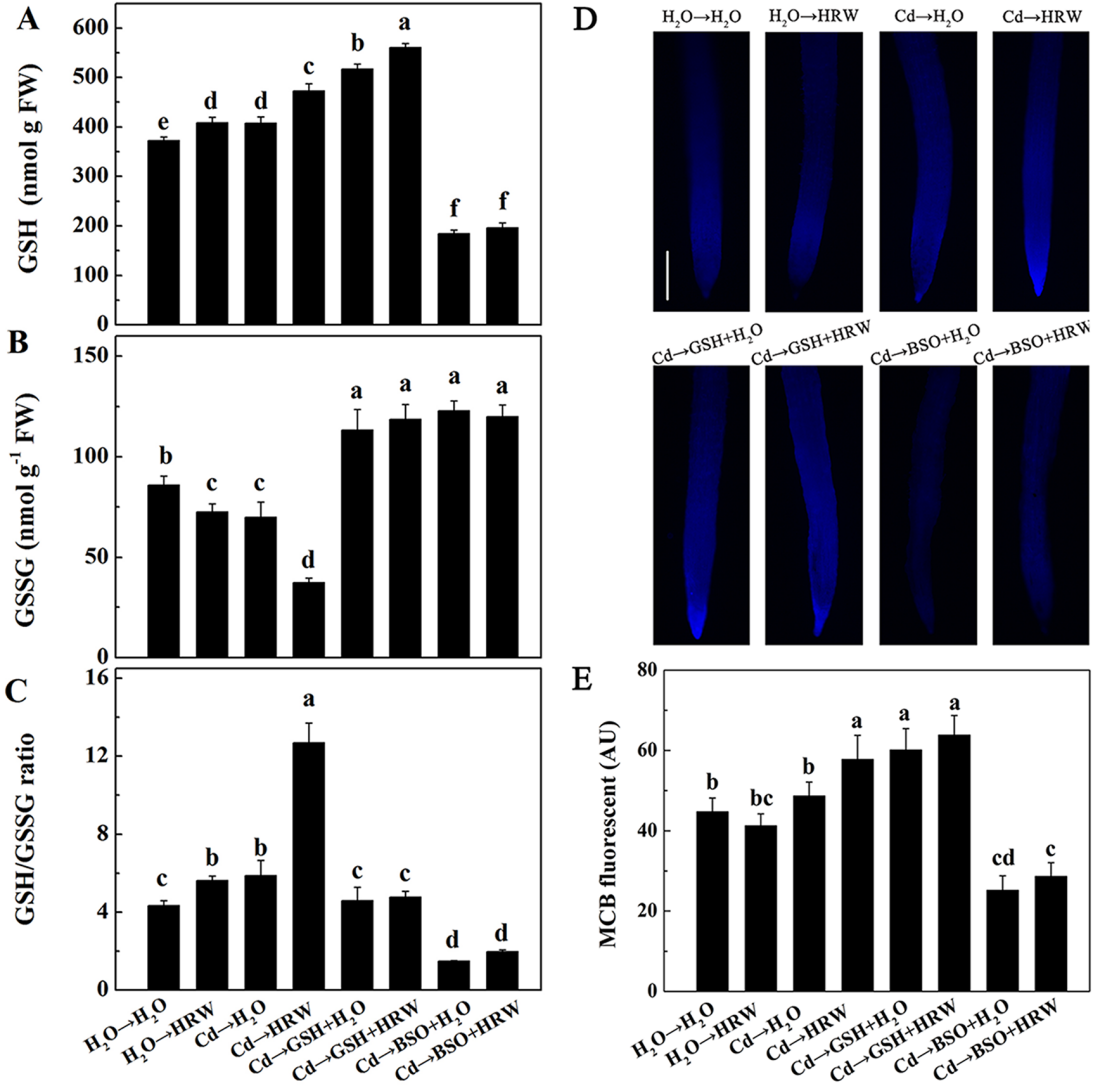
Effects of GSH, BSO and HRW alone or their combinations on GSH pool in the roots of *Brassica campestris* under Cd or none stress. The germinated seeds were grown in nutrient solution for one day and then shifted to solutions with 0 or 50 μM CdCl_2_ for another 12 h. After the removal of Cd stresses, the seedlings were then incubated in solution with 25 μM GSH, 10 μM BSO or 50% saturation of HRW alone or their combinations for 48 h. Concentration of GSH (A), GSSG (B) and the ratio of GSH/GSSG (C) in root tissues were detected at the indicated time points of treatments. Root tissues were loaded with 50 μM monochlorobimane (MCB) for 20 min. Glutathione content was then detected by fluorescence microscope (D). Scale bar = 200 μm. Meanwhile, corresponding intensity of MCB fluorescence (E) was also provided. AU, arbitrary units. Data are means ± SE from three independent experiments. Bars with different letters are significantly different at *P*<0.05 according to Duncan’s multiple range test.

In the non-Cd pretreatments, HRW increased the GSH concentration in the roots of *Brassica campestris* seedlings. The GSH concentration was considerably higher in roots subjected to Cd than in control roots (H_2_O→H_2_O). Compared with Cd treatment alone, GSH concentrations were significantly higher in roots of seedlings post-treated with HRW or GSH, especially exogenous GSH. The highest GSH level (approximately 550 nmol g^-1^) was observed in roots exposed to GSH + HRW treatment after Cd. In addition, GSH synthesis was severely blocked by BSO, whereas this response could be partially reversed by the addition of HRW (Fig 6A). We also noted that post-treatment with HRW alone resulted in a lower GSSG concentration and a much higher GSH/GSSG ratio. In the Cd treatments, the GSSG concentration was higher, although the GSH/GSSG ratio was lower in samples treated with GSH or BSO alone than those treated with HRW or H_2_O. Interestingly, this response could not be reversed by the addition of HRW combined with GSH or BSO (Fig 6B and 6C).

MCB fluorescence analysis was performed to further confirm the above-mentioned GSH changes (Fig 6D and 6E). Control sample roots displayed basal GSH levels (H_2_O→H_2_O). MCB fluorescence was strongly induced in roots post-treated with HRW or GSH. In contrast, the Cd-triggered induction of MCB fluorescence intensity was markedly abolished by the application of BSO. This inhibition was slightly reversed when HRW was added together with BSO.

### Enzyme activity and gene expression analysis of the GSH pool

**Fig 7:**
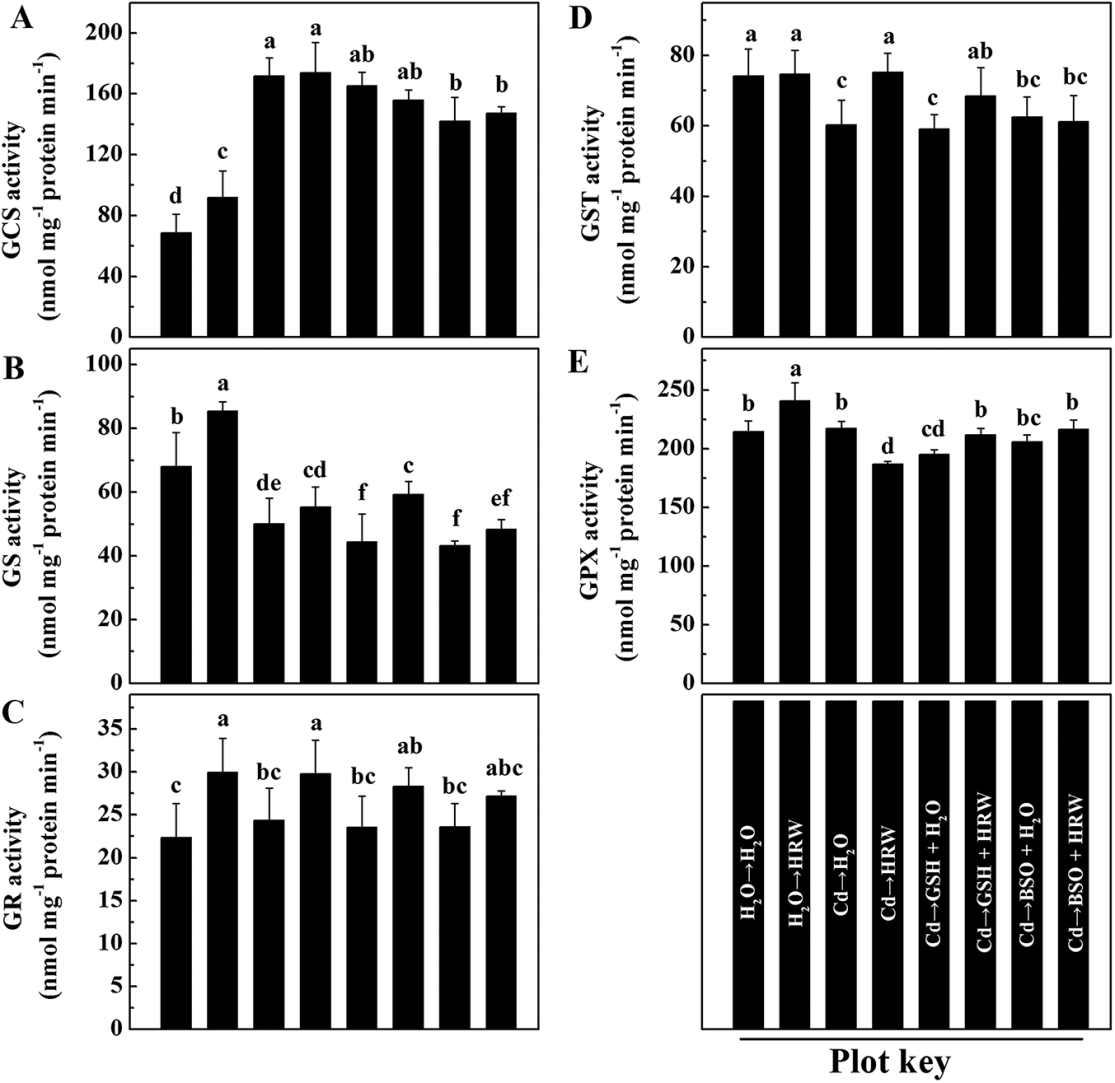
Effects of GSH, BSO and HRW alone or their combinations on gene expression of GCS (A), GS(B), GR(C), GST (D) and GPX (E) activities in the roots of *Brassica campestris* upon Cd or none stress. The germinated seeds were grown in nutrient solution for one day and then shifted to solutions with 0 or 50 μM CdCl_2_ for another 12 h. After the removal of Cd stresses, the seedlings were then incubated in solution with 25 μM GSH, 10 μM BSO or 50% saturation of HRW alone or their combinations for 48 h. Data are means ± SE from three independent experiments. Bars with different letters are significantly different at *P*<0.05 according to Duncan’s multiple range test.

To further study the observed changes in the pool of GSH after exposure to Cd, HRW, GSH and BSO, enzymes and genes involved in its synthesis and/or metabolism, namely GCS, GS, GR, GST, and GPX, were analyzed. In the two Cd-deprived treatments, HRW significantly increased the activities of GCS, GS, GR and GPX but had no effect on GST (Fig 7). Compared to control roots (H_2_O→H_2_O), the activity of GCS was approximately 2-fold higher in roots subjected to 12-h-Cd exposure (Cd→H_2_O). However, the activities of GS and GST were lower, and no influence of Cd on the activities of GR and GPX were observed. Additionally, the activities of GS and GPX were significantly lower in the Cd plus GSH treatment than in the Cd treatment alone (Cd→H_2_O), although the activities of GCS, GR and GST were not affected by GSH treatment. Comparatively, exogenously applied BSO alone significantly reduced the GCS and GS activity but had no apparent effects on the activities of GR, GST or GPX. No obvious impacts of HRW treatment were noted on the activities of GS, GR or GPX in roots subjected to GSH or BSO treatment.

**Fig 8:**
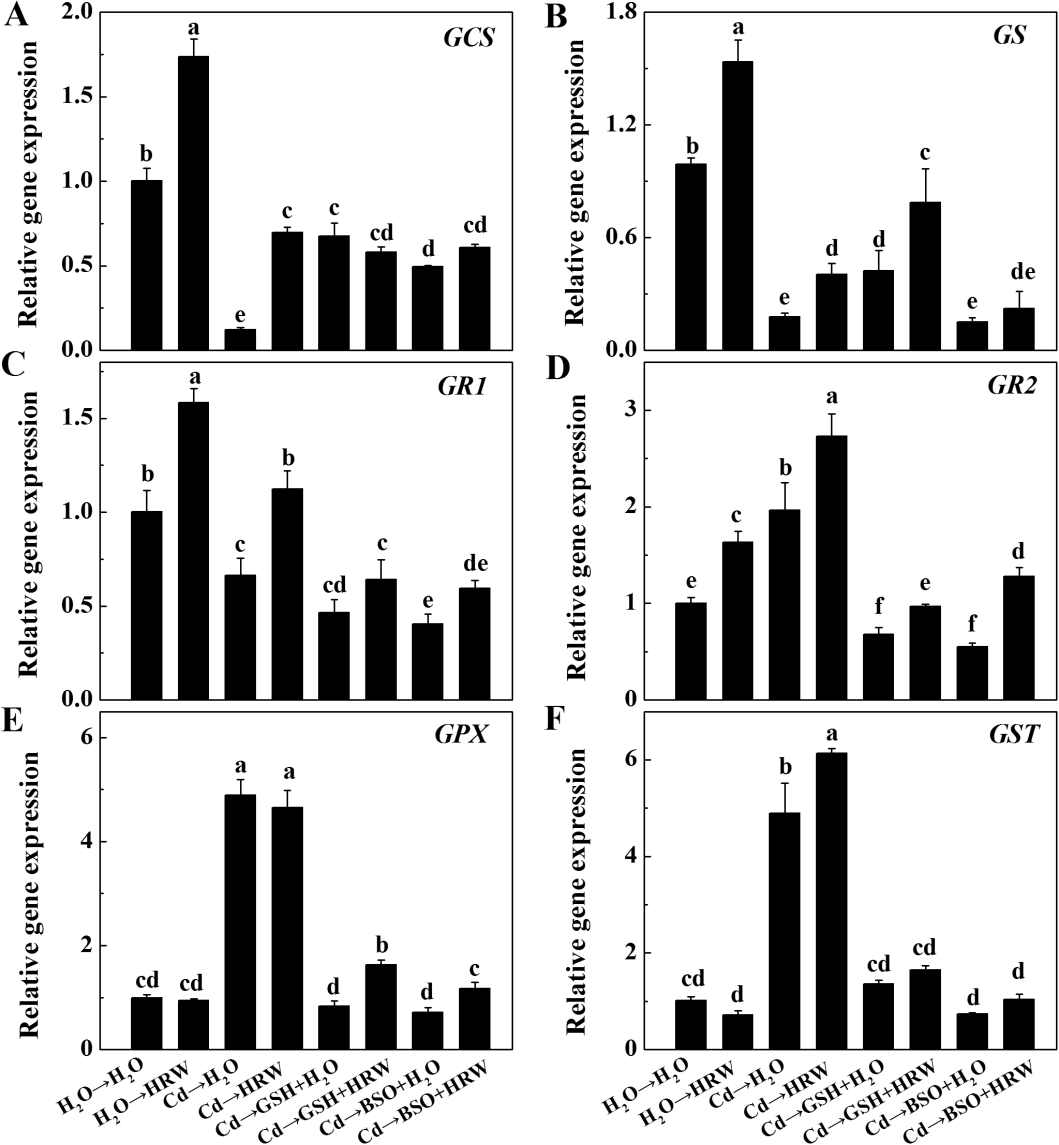
Effects of GSH, BSO and HRW alone or their combinations on gene expression of *GCS*, *GS*, *GR1*, *GR2*, *GPX* and *GST* in the roots of *Brassica campestris* upon Cd or none stress. Expression levels of corresponding genes are presented relative to the control samples, with normalized against expression of two internal reference genes in each sample. Data are means ± SE from three independent experiments. Bars with different letters are significantly different at *P*<0.05 according to Duncan’s multiple range test.

Subsequent gene analysis revealed that seedlings treated with HRW substantially enhanced the transcription levels of genes such as *GCS*, *GS*, *GR1* and *GR2* in the non-Cd pretreatment. A similar HRW-triggered induction of the expression of *GCS*, *GS*, *GR1*, *GR2* and *GST* was also observed in the Cd→HRW treatment compared to the Cd→H_2_O treatment. In treatments with GSH or BSO, the expression of *GCS*, *GS*, *GR1* and *GR2* declined significantly compared with the Cd-free control samples, whereas co-treatment with HRW blocked this decrease to varying degrees (Fig 8A and 8D). The transcript levels of the *GPX* and *GST* genes were increased by pre-treatment with Cd, an effect that was blocked by the addition of GSH or BSO. However, this block was partially reversed by co-treatment with HRW (Fig 8E and 8F).

### Effects of GSH, BSO and HRW on the concentration of AsA in *Brassica campestris* seedling roots

**Fig 9:**
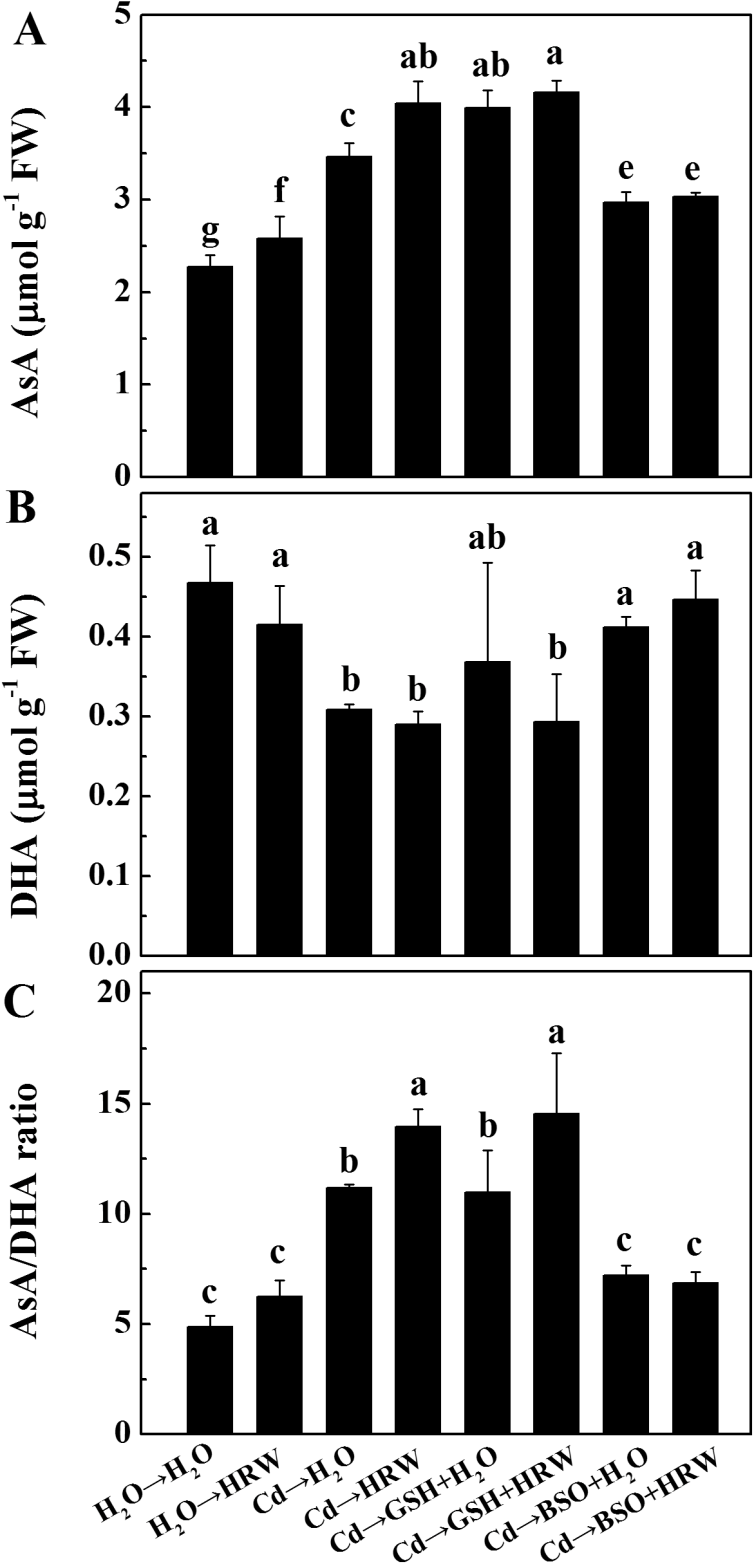
Effects of GSH, BSO and HRW alone or their combinations on ascorbic acid pool (AsA and DHA) in the roots of *Brassica campestris* under Cd or none stress. The germinated seeds were grown in nutrient solution for one day and then shifted to solutions with 0 or 50 μM CdCl_2_ for another 12 h. After the removal of Cd stresses, the seedlings were then incubated in solution with 25 μM GSH, 10 μM BSO or 50% saturation of HRW alone or their combinations for 48 h. Data are means ± SE from three independent experiments. Bars with different letters are significantly different at *P*<0.05 according to Duncan’s multiple range test.

As DHA is derived from AsA via catalysis by DHAR using GSH as an electron donor, we examined the AsA and DHA concentrations in seedling roots. After 12 h of Cd exposure, the AsA concentration was significantly increased after 48 h incubation, and this increase was enhanced by the addition of HRW, GSH, or their combination. When BSO was added alone, these effects were significantly reversed (Fig 9A). In contrast, Cd treatment decreased the DHA concentration, and no rescuing responses occurred when HRW or GSH was added (Fig 9B). Furthermore, the AsA/DHA ratio followed a pattern similar to that of the AsA concentration (Fig 9C).

## Discussion

As the structurally simplest gas in nature, H_2_ gas has been detected in plants under normal growth conditions [19] but accumulates under salt or paraquat stress [8, 9]. Consistent with these observations, we observed that a small amount of H_2_ release was detected in *Brassica campestris* seedlings under control conditions, while an increase was observed under Cd stress (Fig 1). Taken together, these results imply that H_2_ may participate in the regulation of plant tolerance against abiotic stresses, which will be effectively demonstrated by further investigation into the enzymatic source(s) of H_2_ release.

In fact, studies in alfalfa have suggested that pre-treatment with HRW confers increases plant resistance against Cd and aluminum (Al) stresses [10, 11]. However, a question evolved from these reports is proposed here: is HRW-induced resistance against abiotic stress related to the pre-treatment of HRW? Because differences in root elongation between HRW treatment and control had appeared before they were exposed to stresses, which may affect the Cd or Al uptake by roots. In consideration of these observations, we pretreated the seedlings with Cd followed by HRW. Our results illustrated that after 12 h of Cd exposure, seedlings post-treated with HRW exhibited an obvious enhancement in seedling root length and fresh weight compared with the plants treated with Cd alone (Fig 2). Moreover, H_2_-induced recovery of root growth inhibition upon Cd treatment was unlikely to result from increased Cd translocation from roots to shoots, as HRW post-treatment did not lead to greater Cd accumulation in shoots (S1 Fig).

In higher plants, it is well known that Cd toxicity is partially due to induced ROS, which can be partly eliminated by reduced GSH [45, 46]. In this context, GSH concentrations were analyzed to determine whether there is a link between the biosynthesis of GSH, H_2_-induced alleviation of root growth, and oxidative stress triggered by Cd. Our results showed that compared with H_2_O-treated samples, post-treatment with HRW increased the GSH concentration and GSH/GSSG ratio (Fig 3A–3C). It has been suggested that the protective effect of H_2_ might be attributed to its ability to activate both the enzymatic system (e.g., SOD, POD, and APX) and the metabolism of non-enzymatic components such as GSH and AsA [47, 9]. At the same time, it was noticed that there was an obvious fluctuation in the concentration of GSH and GSSG along with the treatment time, which was also observed in other researches [48–50]. In the present study, seedlings were cultivated with a 12 h light and 12 h dark cycle, so the fluctuation might be related to this kind of cycle. However, this speculation needs to be further demonstrated.

Studies in Arabidopsis have demonstrated that an exogenously applied, low concentration of GSH could partially reverse the inhibition in primary root length triggered by drought, salt and Cd stresses [51, 52]. To further verify the function of GSH in H_2_-enhanced Cd tolerance in *Brassica campestris* seedlings, GSH and BSO were exogenously applied. As previously reported in many studies [53], Cd strongly impaired the root elongation of *Brassica campestris* seedlings. However, similar effects occurred in treatments with GSH or HRW, as they both alleviated Cd-induced inhibition of root elongation. In particular, as the phenotype of root elongation induced by HRW disappeared when BSO was added, it is reasonable to assume that GSH is conducive to HRW-induced root elongation to some extent. Our results further illustrate that in addition to an obvious alleviation of root elongation, seedlings post-treated with HRW or GSH exhibited a strong relief of Cd-triggered oxidative damage, supported by both histochemical staining and TBARS analysis. These results are consistent with prior observations [54, 55] and further support the hypothesis that GSH may be involved in H_2_-induced antioxidant behaviors as well as the alleviation of Cd toxicity.

Given the aforementioned results, we have reason to believe that H_2_-mediated remission of Cd stress may be partially due to the regulation of GSH synthesis. Thus, we sought to address the following three questions: 1) How does H_2_ trigger GSH biosynthesis? 2) How do the activities of GSH-metabolism enzymes such as GPX and GST change? 3) Do changes in the GSH level affect the ascorbic acid pool (AsA and DHA)?

A number of metabolic processes in plants can help restore the high GSH-to-GSSH ratio in cells, including increased GSH synthesis, decreased GSH degradation and higher transformation of GSSG to GSH [56–58]. In this report, genetic and pharmacological evidence revealed that GS and GR are mainly responsible for the HRW-induced production of GSH, which was supported by the following three key results. First, GSH production was triggered by HRW or GSH (Fig 6A and 6D) and inhibited by BSO, and this inhibition was markedly reversed by the addition of HRW. Second, the activities of enzymes related to GSH synthesis and redox equilibrium, including GS and GR, were activated by HRW regardless of Cd exposure (Fig 7B and 7C). Third, the downregulation of genes such as *GS*, *GR1* and *GR2* by Cd, GSH or BSO could be reversed to varying degrees by the addition of an HRW solution (Fig 8B and 8D).

In a subsequent test, we observed that GST and GPX isozymatic activities and corresponding transcripts were also partially promoted by HRW (Fig 7D and 7E; Fig 8E and 8F). GST and GPX are two important enzymes for protecting plants against ROS during various stress conditions [59], and GSH acts as the substrate for these enzymes [60]. We therefore deduced that H_2_-mediated enhancement of GSH might at least partially result in the transcripts of *GST* and *GPX* and the corresponding isozymatic activities.

Interestingly, we found a similar effect between the treatments of Cd→GSH and Cd→BSO, both of which inhibited the expression of GSH biosynthesis-related genes, including *GR1*, *GR2*, *GPX* and *GST* (Fig 8C–8F). However, it is clear that the GSH concentrations were much higher under Cd→GSH treatments, but were much lower under Cd→BSO treatments than under Cd-treatment alone (Fig 6A). One possible explanation is that the excessive GSH concentration under exogenous GSH treatments, which mainly resulted from the absorption, could make a feedback inhibition in the expression of GSH biosynthesis-related genes.

As two low molecular weight antioxidants, AsA and GSH are involved in the AsA-GSH cycle [61] and are associated with maintaining cellular redox balance. High ascorbate and glutathione redox ratios are necessary to achieve optimal metabolism and promote tolerance to abiotic stress [62, 63]. In the present study, GSH and AsA concentrations were significantly higher in seedlings post-treated with HRW than in controls. A similar HRW-triggered positive effect was also identified based on the observed AsA/DHA and GSH/GSSG values. These results suggest that the increase in the concentration of AsA might be correlated with a higher GSH level, both of which were enhanced by HRW. Furthermore, the AsA-GSH cycle, an important function in eliminating H_2_O_2_ [64], might also be strengthened by the addition of HRW.

**Fig 10:**
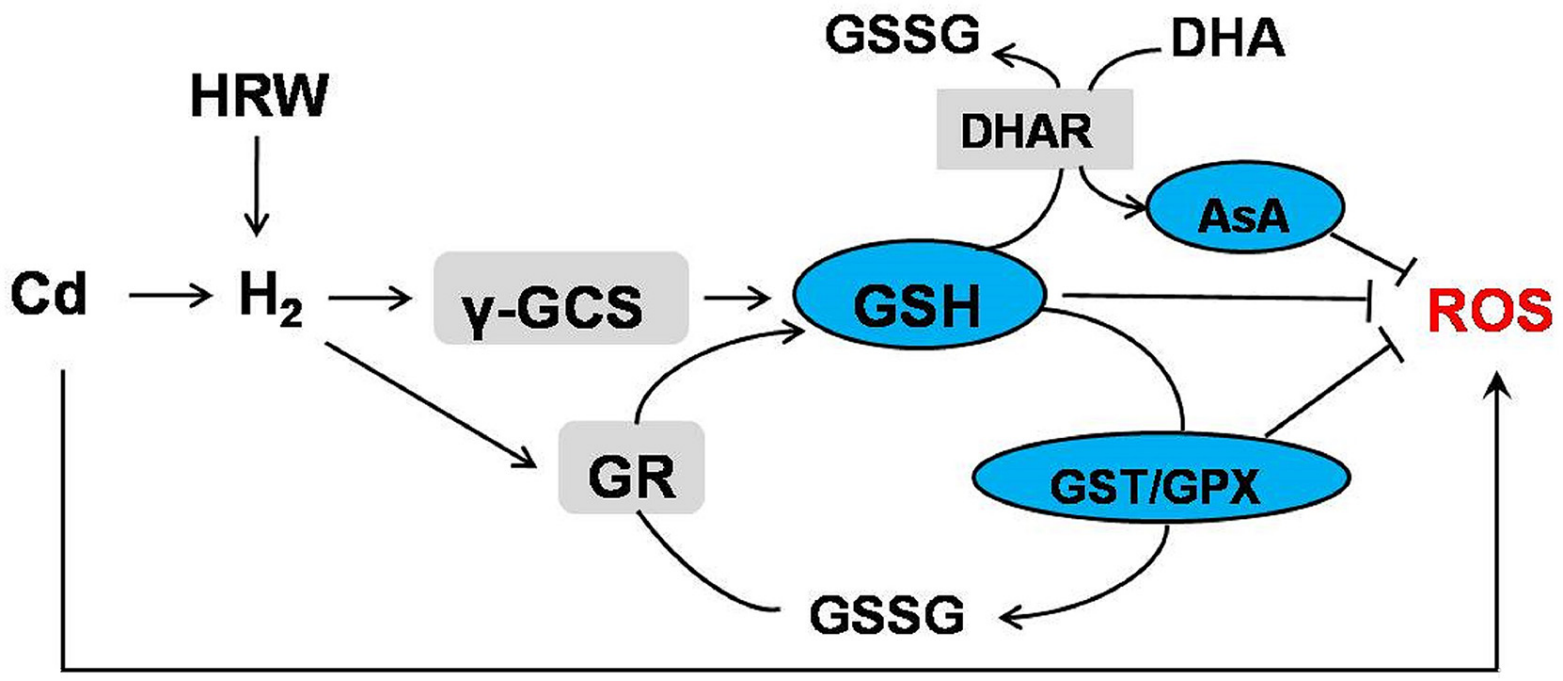
Schematic representation of the pathway induced by cadmium including the participation of H_2_, GSH and AsA.

Taken together, we proposed that H_2_ was able to effectively alleviate Cd stress by exogenously applied HRW, and GSH played an important role in this process. Previous researches reported that γ-GCS and GR led to the enhanced GSH production and the larger total GSH pool size [65]. Furthermore, glutathione showed a strong antioxidant effect against different environment stresses mainly through GSH-dependent regeneration of ascorbate, GSH-dependent peroxide metabolism and GSH itself [66, 67]. In this report, when plants were damaged by Cd, more H_2_ production was induced, followed by promoted γ-GCS and GR activity. This increase of enzyme activities promoted GSH synthesis and reduction from GSSG, respectively. This rise in concentration of GSH was beneficial to the utilization of DHA reductase (DHAR) which thereby reduced DHA to AsA. In addition to the AsA-GSH cycle, other proteins also used GSH as substrate like GPX and GST, and their activities were also strengthened. All these results indicate that H_2_-induced glutathione homeostasis reestablishment exerts a major role in alleviation of Cd stress through ROS detoxification (Fig 10).

## Conclusions

In the present study, greater H_2_ production was observed in seedlings upon exposure to Cd and HRW treatment. Importantly, exogenously applied HRW was able to effectively alleviate the growth inhibition and oxidative damage triggered by Cd stress in Chinese cabbage. It can be concluded that the reestablishment of glutathione homeostasis and a strengthened AsA-GSH cycle could be important reasons why HRW post-treatment improved the tolerance to Cd.

## Supporting Information

S1 Fig Effects of HRW post-treatment on Cd concentration in the seedlings of *Brassica campestris* upon Cd stress. The germinated seeds were grown in nutrient solution for one day and then shifted to solutions with 0 or 50 μM CdCl_2_ for another 12 h. After the removal of Cd stresses, the seedlings were then incubated in solution with H_2_O or 50% saturation of HRW for 6 days. Cd concentration of shoot (A) and root (B) parts of 100 *Brassica campestris* seedlings were determined using atomic absorption spectrophotometry respectively. The bioaccumulation factor (%) presents the ratio of the Cd concentration in shoots to that in roots. Data are means ± SE from three independent experiments. Bars with different letters are significantly different at *P*<0.05 according to Duncan’s multiple range test.
(TIF)

S1 Table The nucleotide sequence of primers used in the RT-PCR. (DOC)

## References

[1] DalCorso G, Farinati S, Maistri S, Furini A (2008) How plants cope with cadmium: staking all on metabolism and gene expression. J Integr Plant Biol 50:1268–1280. 10.1111/j.1744-7909.2008.00737.x 19017114

[2] Zhao XF, Chen L, Rehmani MIA, Wang QS, Wang SH, Hou PF, et al (2008) Effect of nitric oxide on alleviating cadmium toxicity in rice (*Oryza sativa* L.). J Integr Agric 12:1540–1550.

[3] Hassan MJ, Wang F, Ali S, Zhang GP (2005) Toxic effect of cadmium on rice as affected by nitrogen fertilizer form. Plant Soil 277: 359–365.

[4] Ali B, Tao QJ, Zhou YF, Gill RA, Ali S, Rafiq MT, et al (2013) 5-Aminolevolinic acid mitigates the cadmium-induced changes in *Brassica napus* as revealed by the biochemical and ultra-structural evaluation of roots. Ecotoxicol Environ Safety 92: 271–280. 10.1016/j.ecoenv.2013.02.006 23490193

[5] Ali B, Qian P, Jin R, Ali S, Khan M, Aziz R, et al (2014) Physiological and ultra-structural changes in *Brassica napus* seedlings induced by cadmium stress. Biol Plantarum 58: 131–138.

[6] Ali B, Gill AR, Yang S, Gill BM, Farooq AM, Liu D, et al (2015) Regulation of cadmium-induced proteomic and metabolic changes by 5-aminolevulinic acid in leaves of *Brassica napus* L. PLoS ONE 10.1371/journal.pone.0123328 PMC4409391 25909456

[7] Hawrylak-Nowak B, Dresler S, Matraszek R (2015) Exogenous malic and acetic acids reduce cadmium phytotoxicity and enhance cadmium accumulation in roots of sunflower plants. Plant Physiology and Biochemistry 94:225–234. 10.1016/j.plaphy.2015.06.012 26115548

[8] Xie Y, Mao Y, Lai D, Zhang W, Shen W (2012) H_2_ enhances Arabidopsis salt tolerance by manipulating ZAT10/12-mediated antioxidant defence and controlling sodium exclusion. PLoS One 7: e49800 10.1371/journal.pone.0049800 23185443 PMC3504229

[9] Jin Q, Zhu K, Cui W, Xie Y, Han B, Shen W (2013) Hydrogen gas acts as a novel bioactive molecule in enhancing plant tolerance to paraquat-induced oxidative stress via the modulation of heme oxygenase-1 signaling system. Plant Cell Environ 36:956–969. 10.1111/pce.12029 23094798

[10] Chen M, Cui W, Zhu K, Xie Y, Zhang C, Shen W (2014) Hydrogen-rich water alleviates aluminum-induced inhibition of root elongation in alfalfa via decreasing nitric oxide production. J Hazard Mater 267:40–47. 10.1016/j.jhazmat.2013.12.029 24413050

[11] Cui W, Gao C, Fang P, Lin G, Shen W (2013) Alleviation of cadmium toxicity in *Medicago sativa* by hydrogen-rich water. J Hazard Mater 260:715–724. 10.1016/j.jhazmat.2013.06.032 23846121

[12] Hu H, Li P, Wang Y, Gu R (2014) Hydrogen-rich water delays postharvest ripening and senescence of kiwifruit. Food Chem 156:100–109. 10.1016/j.foodchem.2014.01.067 24629944

[13] Lin Y, Zhang W, Qi F, Cui W, Xie Y, Shen W (2014) Hydrogen-rich water regulates cucumber adventitious root development in a heme oxygenase-1/carbon monoxide-dependent manner. J Plant Physiol 171:1–8. 10.1016/j.jplph.2013.08.009 24331413

[14] Su N, Wu Q, Liu Y, Cai J, Shen W, Xia K, et al (2014) Hydrogen-rich water reestablishes ROS homeostasis but exerts differential effects on anthocyanin synthesis in two varieties of radish sprouts under UV-A irradiation. J Agric Food Chem 62: 6454–6462. 10.1021/jf5019593 24955879

[15] Melis A, Happe T (2001) Hydrogen production. Green algae as a source of energy. Plant Physiol 127:740–748. 11706159 PMC1540156

[16] Meyer J (2007) [FeFe] hydrogenases and their evolution: a genomic perspective. Cell Mol Life Sci 64:1063–1084. 17353991 10.1007/s00018-007-6477-4 PMC11136429

[17] Gaffron H (1939) Reduction of carbon dioxide with molecular hydrogen in green algae. Nature 143:204–205.

[18] Renwick GM, Giumarro C, Siegel SM (1964) Hydrogen metabolism in higher plant. Plant Physiol 39:303–306. 16655917 10.1104/pp.39.3.303 PMC550076

[19] Torres V, Ballesteros A, Fernández VM, Nunez M (1984) Expression of hydrogenase activity in cereals. Ann N Y Acad Sci 434:296–298.

[20] Cho UH, Seo NH (2005) Oxidative stress in Arabidopsis thaliana exposed to cadmium is due to hydrogen peroxide accumulation. Plant Sci 168:113–120.

[21] Romero-Puertas MC, Rodríguez-Serrano RM, Corpas FJ, Gómez M, Del Río LA, Sandalio LM (2004) Cadmium-induced subcellular accumulation of O_2_·^-^and H_2_O_2_ in pea leaves. Plant Cell Environ 27:1122–1134.

[22] Mellado M, Contreras RA, González A, Dennett G, Moenne A (2012) Copper-induced synthesis of ascorbate, glutathione and phytochelatins in the marine alga *Ulva compressa* (Chlorophyta). Plant Physiol Bioch 51:102–108. 10.1016/j.plaphy.2011.10.007 22153245

[23] Wu FB, Chen F, Wei K, Zhang GP (2004) Effect of cadmium on free amino acid, glutathione and ascorbic acid concentrations in two barley genotypes (*Hordeum vulgare* L.) differing in cadmium tolerance. Chemosphere 57: 447–454. 15350406 10.1016/j.chemosphere.2004.06.042

[24] Foyer CH, Noctor G (2005) Redox homeostasis and antioxidant signaling: a metabolic interface between stress perception and physiological responses. Plant Cell 17:1866–1875. 15987996 10.1105/tpc.105.033589 PMC1167537

[25] Millar AH, Mittova V, Kiddle G, Heazlewood JL, Bartoli CG, Theodoulou FL, et al (2003) Control of ascorbate synthesis by respiration and its implications for stress responses. Plant Physiol 133: 443–447. 14555771 10.1104/pp.103.028399 PMC1540335

[26] Márquez-García B, Horemans N, Torronteras R, Córdoba F (2012) Glutathione depletion in healthy cadmium-exposed *Erica andevalensis* . Environ Exp Bot 75:159–166.

[27] Pomponi M, Censi V, Girolamo VD, Paolis AD, Toppi LS, Aromolo R, et al (2006) Overexpression of Arabidopsis phytochelatin synthase in tobacco plants enhances Cd^2+^ tolerance and accumulation but not translocation to the shoot. Planta 223:180–190. 16133212 10.1007/s00425-005-0073-3

[28] Noctor G, Mhamdi A, Chaouch S, Han Y, Neukermans J, Marquez-Garcia B, et al (2012) Glutathione in plants: an integrated overview. Plant Cell Environ 35: 454–484. 10.1111/j.1365-3040.2011.02400.x 21777251

[29] Wu Q, Su N, Cai J, Shen Z, Cui J (2015) Hydrogen-rich water enhances cadmium tolerance in Chinese cabbage by reducing cadmium uptake and increasing antioxidant capacities. J plant physiol 175:174–182. 10.1016/j.jplph.2014.09.017 25543863

[30] Chen J, Jiang H, Hsieh EJ, Chen HY, Chien CT, Hsieh HL, et al (2012) Drought and salt stress tolerance of an Arabidopsis glutathione S-Transferase U17 knockout mutant are attributed to the combined effect of glutathione and abscisic acid. Plant Physiol 158:340–351. 10.1104/pp.111.181875 22095046 PMC3252094

[31] Wei S, Zhou Q (2004) Identification of weed species with hyperaccumulative characteristics of heavy metals. Prog Nat Sci 14:495–503.

[32] Liu J, Zhou Q, Sun T, Ma L, Wang S (2008) Growth responses of three ornamental plants to Cd and Cd-Pb stress and their metal accumulation characteristics. J Hazard Mater 151:261–267. 17869419 10.1016/j.jhazmat.2007.08.016

[33] Sung C, Hong J (2010) Sodium nitroprusside mediates seedling development and attenuation of oxidative stresses in Chinese cabbage. Plant Biotech Rep 4:243–251.

[34] Yamamoto Y, Kobayashi Y, Matsumoto H (2001) Lipid peroxidation is an early symptom triggered by aluminum, but not the primary cause of elongation inhibition in pea roots. Plant Physiol 125:199–208. 11154329 10.1104/pp.125.1.199 PMC61002

[35] Müller M, Kok LJD, Weidner W, Tausz M (2002) Differential effects of H_2_S on cytoplasmic and nuclear thiol concentrations in different tissues of *Brassica* roots. Plant Physiol Biochem 40:585–589.

[36] Han B, Yang Z, Xie Y, Nie L, Cui J, Shen W (2014) Arabidopsis HY1 confers cadmium tolerance by decreasing nitric oxide production and improving iron homeostasis. Mol Plant 7: 388–403. 10.1093/mp/sst122 23974911

[37] Xia X, Zhang Y, Wu J, Wang J, Zhou Y, Shi K, et al (2009) Brassinosteroids promote metabolism of pesticides in cucumber. J Agric Food Chem 57:8406–8413. 10.1021/jf901915a 19694443

[38] Cakmak I, Marschner H (1992) Magnesium deficiency and high light intensity enhance activities of superoxide dismutase, ascorbate peroxidase, and glutathione reductase in bean leaves. Plant Physiol 98:1222–1227. 16668779 10.1104/pp.98.4.1222 PMC1080336

[39] Pailan RV, Yallapragada PR, Thatipaka SDR (2012) Response of glutathione system and carotenoids to sublethal copper in the postlarvae of Penaeus indicus. Ecotox Environ Safe 75: 127–133. 10.1016/j.ecoenv.2011.08.023 21944956

[40] Jez JM, Cahoon RE (2004) Kinetic mechanism of glutathione synthase from *Arabidopsis thaliana* . J Biol Chem 279:42726–42731. 15302873 10.1074/jbc.M407961200

[41] Arisi ACM, Noctor G, Foyer CH, Jouanin L (1997) Modifications of thiol contents in poplars (*Populus tremula*×*P*. *alba*) overexpressing enzymes involved in glutathione synthesis. Planta 203:362–372. 9431683 10.1007/s004250050202

[42] Rahman I, Kode A, Biswas SK (2006) Assay for quantitative determination of glutathione and glutathione disulfide levels using enzymatic recycling method. Nat Protoc 1; 3159–3165. 17406579 10.1038/nprot.2006.378

[43] Srivastava PK, Singh VP, Prasad SM (2012) Compatibility of ascorbate-glutathione cycle enzymes in cyanobacteria against low and high UV-B exposures, simultaneously exposed to low and high doses of chlorpyrifos. Ecotox Environ Safe 83:79–88. 10.1016/j.ecoenv.2012.06.009 22784817

[44] Xiao D, Zhang N, Zhao J, Bonnema G, Hou X (2012) Validation of reference genes for real-time quantitative PCR normalisation in non-heading Chinese cabbage. Funct Plant Biol 39:342–350. 10.1071/FP11246 32480786

[45] Foyer CH, Theodoulou FL, Delrot S (2001) The functions of inter- and intracellular glutathione transport systems in plants. Trends Plant Sci 6:486–492. 11590068 10.1016/s1360-1385(01)02086-6

[46] Sharma SS, Dietz KJ (2009) The relationship between metal toxicity and cellular redox imbalance. Trends Plant Sci 14:43–50. 10.1016/j.tplants.2008.10.007 19070530

[47] Ohsawa I, Ishikawa M, Takahashi K, Watanabe M, Nishimaki K, Yamagata K, et al (2007) Hydrogen acts as a therapeutic antioxidant by selectively reducing cytotoxic oxygen radicals. Nat Med 13: 688–694. 17486089 10.1038/nm1577

[48] Chen H, Huang C, Huang G, Chow T, Lin Y (2013) NADPH oxidase inhibitor diphenyleneiodonium and reduced glutathione mitigate ethephon-mediated leaf senescence, H2O2 elevation and senescence-associated gene expression in sweet potato (*Ipomoea batatas*). J Plant Physiol 170: 1471–1483. 10.1016/j.jplph.2013.05.015 23834930

[49] Chen F, Wang F, Wu F, Mao W, Zhang G, Zhou M (2010) Modulation of exogenous glutathione in antioxidant defense system against Cd stress in the two barley genotypes differing in Cd tolerance. Plant Physiol Bioch 48: 663–672. 10.1016/j.plaphy.2010.05.001 20605723

[50] Wang J, Zeng Q, Zhu J, Liu G, Tang H (2013) Dissimilarity of ascorbate-glutathione (AsA-GSH) cycle mechanism in two rice (*Oryza sativa* L.) cultivars under experimental free-air ozone exposure. Agr Ecosyst Environ 165: 39–49.

[51] Huang J, Zhang Y, Peng J, Zhong C, Yi H, David WO, et al (2012) Fission yeast HMT1 lowers seed cadmium through phytochelatin-dependent vacuolar sequestration in Arabidopsis. Plant Physiol 158:1779–1788. 10.1104/pp.111.192872 22319073 PMC3320185

[52] Chen J, Jiang H, Hsieh EJ, Chen H, Chien CT, Hsieh HL, et al (2012) Drought and salt stress tolerance of an Arabidopsis glutathione S-transferase U17 knockout mutant are attributed to the combined effect of glutathione and abscisic acid. Plant Physiol 158: 340–351. 10.1104/pp.111.181875 22095046 PMC3252094

[53] Podazza G, Arias M, Prado FE (2012) Cadmium accumulation and strategies to avoid its toxicity in roots of the citrus rootstock Citrumelo. J Hazard Mater 216:83–89. 10.1016/j.jhazmat.2012.02.031 22410717

[54] Xu S, Zhu S, Jiang Y, Wang N, Wang R, Shen W, et al (2013) Hydrogen-rich water alleviates salt stress in rice during seed germination. Plant Soil 370:47–57.

[55] Chen F, Wang F, Wu F, Mao W, Zhang G, Zhou M (2010) Modulation of exogenous glutathione in antioxidant defense system against Cd stress in the two barley genotypes differing in Cd tolerance. Plant Physiol Biochem 48:663–672. 10.1016/j.plaphy.2010.05.001 20605723

[56] Noctor G, Foyer CH (1998) Ascorbate and glutathione: keeping active oxygen under control. Annu Rev Plant Physiol Plant Mol Biol 49: 249–279. 15012235 10.1146/annurev.arplant.49.1.249

[57] Aravind P, Prasad MNV (2005) Cadmium and zinc interactions in a hydroponic system using *Ceratophylum demersum* L.: adaptive ecophysiology, biochemistry and molecular toxicology. Braz J Plant Physiol 17:3–20.

[58] Noctor G, Queval G, Mhamdi A, Chaouch S, Foyer CH (2011) Glutathione. In: the Arabidopsis book. Am Soc Plant Biol 10.1199/tab.0142 PMC3267239 22303267

[59] Anjum NA, Ahmad I, Mohmood I, Pacheco M, Duarte AC, Pereira E, et al (2012) Modulation of glutathione and its related enzymes in plants’ responses to toxic metals and metalloids. Environ Exp Bot 75:307–324.

[60] Oost RVD, Beyer J, Vermeulen NP (2003) Fish bioaccumulation and biomarkers in environmental risk assessment: a review. Environ Toxicol Pharmacol 13:57–149. 21782649 10.1016/s1382-6689(02)00126-6

[61] Drazkiewicz M,. Polit ES, Krupa Z (2003) Response of the ascorbate-glutathione cycle to excess copper in *Arabidopsis thaliana* (L.). Plant Sci 164:195–202.

[62] Mittler R (2002) Oxidative stress, antioxidants and stress tolerance. Trends Plant Sci 7:405–410. 12234732 10.1016/s1360-1385(02)02312-9

[63] Foyer CH, Noctor G (2005) Redox homeostasis and antioxidant signaling: a metabolic interface between stress perception and physiological responses. Plant Cell 17: 1866–1875. 15987996 10.1105/tpc.105.033589 PMC1167537

[64] Rao MV, Beverley AH, Douglas PO (1995) Amelioration of ozone induced oxidative damage in wheat plants grown under high carbon dioxide. Plant Physiol 109:421–432. 12228603 10.1104/pp.109.2.421 PMC157604

[65] Dietz KJ, Jacob S, Oelze ML, Laxa M, Tognetti V, de Miranda SM, et al (2006) The function of peroxiredoxins in plant organelle redox metabolism. J Exp Bot 57:1697–1709. 16606633 10.1093/jxb/erj160

[66] Diaz-Vivancos P, Dong YP, Ziegler K, Markovic J, Pallardo F, Pellny TK, et al (2010) Recruitment of glutathione into the nucleus during cell proliferation adjusts whole cell redox homeostasis in Arabidopsis thaliana and lowers the oxidative defence shield. Plant J 64: 825–838. 10.1111/j.1365-313X.2010.04371.x 21105929

[67] Foyer CH, Noctor G (2011) Ascorbate and Glutathione: The Heart of the Redox Hub. Plant Physiol 155: 2–18. 10.1104/pp.110.167569 21205630 PMC3075780

