# Supplementary material for: Cadmium-Induced Hydrogen Accumulation Is Involved in Cadmium Tolerance in Brassica campestris by Reestablishment of Reduced Glutathione Homeostasis
Source: PLoS One. 2015 Oct 7;10(10):e0139956. doi: 10.1371/journal.pone.0139956 (PMC4596834; doi:10.1371/journal.pone.0139956)
Supplement: S1 Table — (DOC) [file pone.0139956.s002.doc]

**Table S1** The nucleotide sequence of primers used in the RT-PCR

| cDNA | Primer forward | Primer reverse |
| --- | --- | --- |
| Actin | 5'-GGAGCTGAGAGATTCCGTTG-3' | 5'-GAACCACCACTGAGGACGAT-3' |
| GCS | 5'-TGCAACCTATAGCAACGGCT-3' | 5'-AAGGTAGCATTCCTGTGCGG-3' |
| GS | 5'-TGGAACCCTTACTGTTGGGG-3' | 5'-ACTTGACAGCTGAGGACTGC-3' |
| GR1 | 5'-GACTTCCAGACCGGGTGTTT-3' | 5'-GCCAGCTTTTACAGCAACCC-3' |
| GR2 | 5'-CACCATTAGCTGTGGTGGGT-3' | 5'-TATCCTGCCGTCCAGAAATG-3' |
| GPX | 5'-TGGTTTCTCCTGACGGCAAA-3' | 5'-GAAGCCTGTCCCAACAGAGT-3' |
| GST | 5'-GGCCGAGCAGACAAGAGAAT-3' | 5'-CGGAATCATACTTCCGGCGA-3' |
